# Supplementary material for: Inflammatory factors and risk of lung adenocarcinoma: a Mendelian randomization study mediated by blood metabolites
Source: Front Endocrinol (Lausanne). 2024 Aug 27;15:1446863. doi: 10.3389/fendo.2024.1446863 (PMC11384989; doi:10.3389/fendo.2024.1446863)
Supplement: Supplementary file 1 [file DataSheet1.zip › supplementary files/S2/GCST90200256/sensitivity-analysis.pdf]

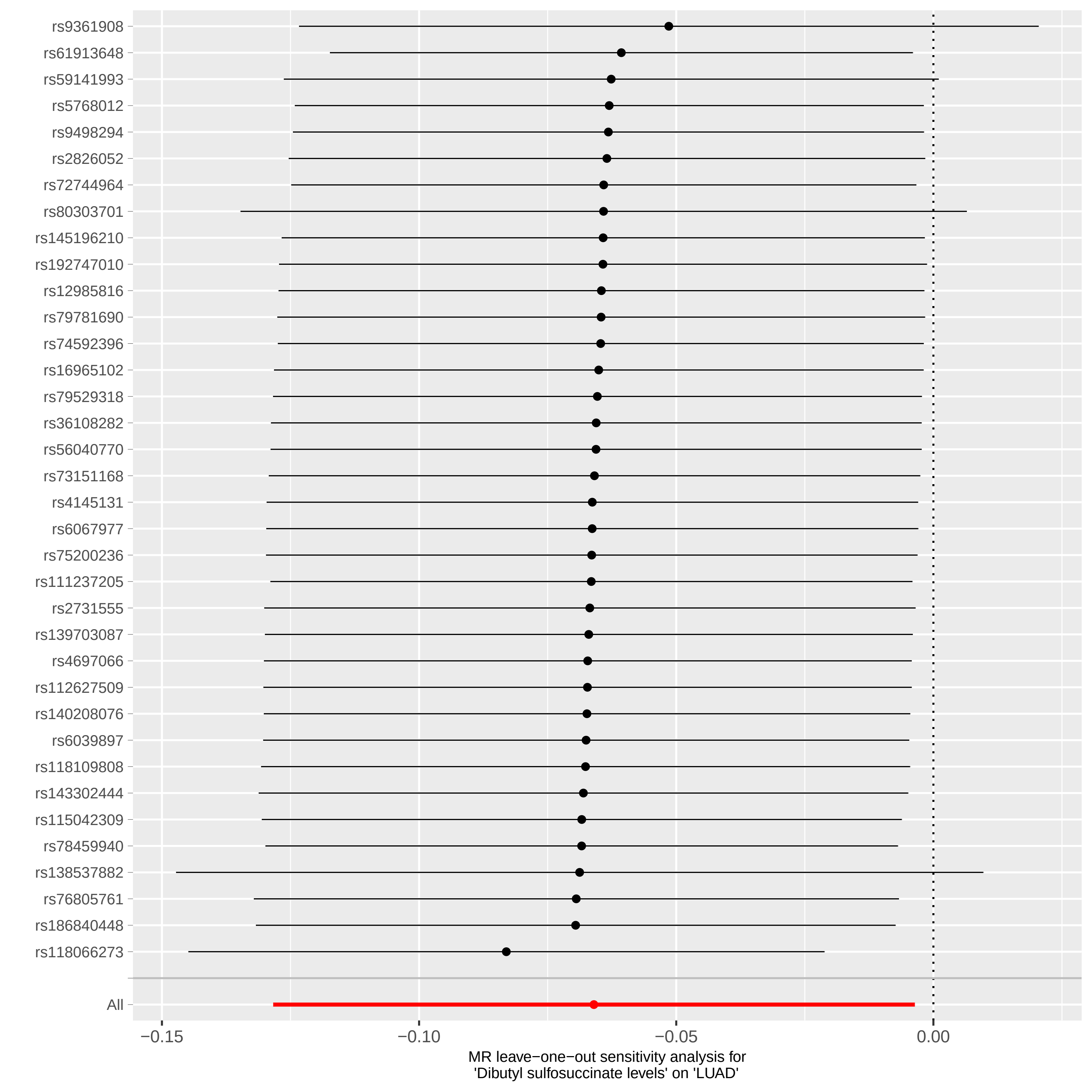
rs9361908
rs61913648
rs59141993
rs5768012
rs9498294
rs2826052
rs72744964
rs80303701
rs145196210
rs192747010
rs12985816
rs79781690
rs74592396
rs16965102
rs79529318
rs36108282
rs56040770
rs73151168
rs4145131
rs6067977
rs75200236
rs111237205
rs2731555
rs139703087
rs4697066
rs112627509
rs140208076
rs6039897
rs118109808
rs143302444
rs115042309
rs78459940
rs138537882
rs76805761
rs186840448
rs118066273
All
−0.15
−0.10
−0.05
0.00
MR leave−one−out sensitivity analysis for
'Dibutyl sulfosuccinate levels' on 'LUAD'
